# Grand challenges for biological engineering

**DOI:** 10.1186/1754-1611-3-16

**Published:** 2009-09-22

**Authors:** Jeong-Yeol Yoon, Mark R Riley

**Affiliations:** 1 Department of Agricultural and Biosystems Engineering, The University of Arizona, Tucson, Arizona 85721-0038, USA

## Abstract

Biological engineering will play a significant role in solving many of the world's problems in medicine, agriculture, and the environment. Recently the U.S. National Academy of Engineering (NAE) released a document "Grand Challenges in Engineering," covering broad realms of human concern from sustainability, health, vulnerability and the joy of living. Biological engineers, having tools and techniques at the interface between living and non-living entities, will play a prominent role in forging a better future. The 2010 Institute of Biological Engineering (IBE) conference in Cambridge, MA, USA will address, in part, the roles of biological engineering in solving the challenges presented by the NAE. This letter presents a brief outline of how biological engineers are working to solve these large scale and integrated problems of our society.

## Grand challenges for engineering

The U.S. National Academy of Engineering (NAE) has recently published a document presenting "Grand Challenges for Engineering," available at [1]. This list was proposed by leading engineers and scientists from around the world at the request of the U.S. National Science Foundation (NSF). Fourteen topics were selected for these grand challenges, and at least seven can be addressed using the tools and methods of biological engineering.

1. Develop carbon sequestration methods

2. Manage the nitrogen cycle

3. Provide access to clean water

4. Advance health informatics

5. Engineer better medicines

6. Reverse-engineer the brain

7. Engineer the tools of scientific discovery

Below we describe a few of ways that biological engineering impacts these challenges.

## Develop carbon sequestration methods

Carbon dioxide (CO_2_) has been identified as a prime contributor to global warming, and efforts have begun in carbon sequestration which involves capturing and storing CO_2_. Depleted oil and gas fields have often been suggested for storing this captured CO_2 _due to the large volume of seemingly inert materials. Sedimentary brine formations (deep underground) have also been suggested as storage repositories.

Both methods are less than ideal as they do not depend on natural, above ground methods of our planet: terrestrial sequestration. As continuous deforestation is undermining our planet's ability to sequester CO_2_, a sustainable, ecologically intensive agroecosystem is warranted. This system should mimic those of the natural floral of our planet, is likely to provide greater throughput (although not greater capacity than the underground methods) [2,3] and can interface with well developed methods that simultaneously generate desirable products. One example of such a process is the use of algae. CO_2 _can be captured from smokestacks and used along with municipal wastewater to grow algae that can be later processed into transportation fuels. Engineering challenges in this specific solution include, for example, increasing surface area for capturing CO_2 _from smokestacks, bioreactor design to maximize the biofuel yield from algae, synthetic biology to enhance algae's carbon sequestration potential and to generate high value products efficiently, ecological engineering to design interfaces between the system and externalities, and optimizing benefit-to-cost ratio for the entire process. Such an approach does not eliminate the release of CO_2_, but does permit a true recycling process. Advances are still needed in the areas of strain development (using traditional and advanced methods), harvesting and dewatering, and separation of desired compounds in order for such an approach to be economically competitive.

## Manage the nitrogen cycle

Nitrogen is an essential component of proteins and DNA/RNA, consequently needed by all living things. Unfortunately, atmospheric nitrogen cannot be readily used to synthesize proteins and DNA. Nitrogen fixation is required to convert atmospheric nitrogen into ammonia, nitrate or nitrogen dioxide (fixed nitrogen) by bacteria, which can then be converted into amino acids and nucleotides, and finally into proteins and DNA. Nitrogen accumulation in rivers and streams, delivered by runoff from agricultural overuse of nitrogen fertilizers, threatens fish and other aquatic species.

Modifying plants and cropping systems is a good starting point to increase nitrogen use efficiency, which should reduce both nitrogen fertilizer demand and water quality impacts. Macroscopic analysis of nitrogen management/cycle should be added on top of this to manage and optimize the complete nitrogen cycle. Nutrient management in rivers and streams is difficult due to many factors including the spatial and temporal variability of nitrogen introduction, water quality and quantity, and algal growth responses. Seasonality can play a significant role in the sensitivity to nutrients and so managing rivers and streams for nutrients will require methods for measuring *in situ *responses and sensitivities to nutrient enrichment [4]. Nitrogen metabolism by microbial communities appears to represent a tradeoff between growth in low nitrogen environments and stress resistance in high ammonia environments [5]. A deeper understanding of the nitrogen cycle is required both to reduce fertilizer use and to manage its environmental impact. A systematic study over a relatively large system (e.g., for an entire river) is warranted to find out an optimal solution in managing this nitrogen cycle. Both macroscopic models with lumped parameter analysis and numerical simulation over large number of grids can be used. Unmanned sensor networks (e.g., ion-selective electrodes) may be useful in studying this cycle over a multitude of conditions.

## Provide access to clean water

The Grand Challenge document addresses the issue of water supply by quoting Samuel Taylor, "water, water, everywhere, nor any drop to drink." The lack of access to clean water results in more deaths in the world than does war. A recent U.N. report warns "overcoming the crisis in water and sanitation is one of the greatest human development challenges of the early 21st century." Desalination, extracting the salt from seawater, is coming closer to being an economical and practical source of potable water in coastal regions, but is considered to be still too costly.

Traditional approaches such as improving irrigation systems and plant water use efficiency should provide some solutions to our water crisis. Again, there are roles for both systematic approach of ecological engineering and synthetic biology of plants.

The most practical approach, at least in the short term, appears to involve sewage treatment and recycling of wastewater. Effective methods rely on biological processing but at the same time limitations arise due to the presence of biological components (pathogens and parasites, endocrine disrupting compounds, and the like). The potential danger of recycling is exacerbated by the lack of real-time monitoring capability to track the concentrations of compounds and organisms that could impact health and environment.

Methods capable of rapid and very sensitive detection are warranted. Lab-on-a-chip systems may meet both requirements utilizing the recent advancements in optics technologies [6,7] and through full automation. Such systems could be integrated into drinking water distribution systems to ensure that drinking water is safe to consume [8]. New antimicrobial compounds, surfaces, and methods may also improve the safety of our natural resources and mitigate the spread of waterborne infections [9].

## Advance health informatics

There is an urgent need for improving the methods for maintaining health care patient records, which are currently a mixture of paper-based and computer-based information. Medical information is often gained outside of the physician's office but cannot currently be integrated into patient records without a visit to the doctor. Technical platforms for collecting and transmitting such data are already available (i.e. a mobile phone with internet access), but must be integrated with sensors, expert systems, and information science.

Despite much laboratory success, few health-related biosensors have transitioned into successful commercial products, although glucose sensors have gained a solid footing. Once widespread challenges of sample pre-processing, filtration, and multiple reagent additions are resolved, a set of biosensors may be integrated into a microfluidic network of channels (i.e., lab-on-a-chip) into a mobile health care device ("lab-on-a-phone") [10]. Such systems could enable networking of health informatics both for preservation of an individual's health but also to monitor for public health emergencies including outbreaks in the spread of disease.

## Engineer better medicines

There is growing recognition for patient-specific medicines to address variability in susceptibility to disease and response to treatment modalities. Advances in genetic and genomic sciences combined with patient clinical data may permit development of optimally tailored preventative measures and treatments. Specific challenges include not only improving drug efficacy with low side effects, but also reducing the time for development, improved safety analysis, and lowered final cost [11].

Tissue engineering plays an important role here not only in replacing diseased and damaged tissue, but also for development and high throughput screening of medications. Stem cell development methods, including inducible pluripotent stem cells, open new avenues for the rigorous testing needed before widespread adoption of any new medical procedures or compounds [12]. Physicians may desire somatic (i.e., adult) stem cells over embryonic ones to get around both potential ethics and availability issues.

## Reverse-engineer the brain

Improved understanding of brain physiology and function will serve the dual roles of providing solutions to many brain disorders and may improve how computers can emulate human intelligence. Artificial intelligence, speech recognition and machine vision systems have made significant advances in part by mimicking what has been learned in how higher organisms process information. To quote the Grand Challenge document: "Neurological disorders may someday be circumvented by technological innovations that allow wiring of new materials into our bodies to do the jobs of lost or damaged nerve cells. Implanted electronic devices could help victims of dementia to remember, blind people to see, and crippled people to walk."

Neuronal regeneration, once thought to be impossible, has been demonstrated for recovery of function following peripheral nerve lesions by guiding axons back to their original target end-organs [13]. Recent studies of spinal cord damage have demonstrated that loss of function is largely irreversible, except at the earliest stages. After breach of the membranes of vulnerable cells adjacent to an injury site, damage to the nervous system leads to large bioelectric currents that cause further harm in the early stages of neurotrama [14]. Mechanical damage may also be repaired following spontaneous reassembly of cell membranes made possible by the action of targeted hydrophilic polymers which seal the compromised portion of the plasmalemma, and allow the lipidic core of the compromised membranes to resolve into each other [15]. These new findings will help establish improved procedures and equipment necessary for neurosurgery, implanted neural sensor that can accept the body's neural signals, and internal or external devices to replace damaged eyes or legs, etc. Biocompatibility study should also follow for these sensors and devices.

## Engineer the tools of scientific discovery

Making new tools for broader scientific discovery is the goal of much biological engineering research and development leading to advances in imaging, biomolecular analysis, and environmental monitoring. Biological engineering also applies systems approaches which connect across application scales. One example is synthetic biology which is moving forward due in part to the development of reusable, standard, and interchangeable genetic elements, "BioBricks" [16,17]. As with many life science endeavors, standardized tools, techniques, and units of measurement require further refinement so as to facilitate transformation of information across laboratories.

## Concluding remarks

Described above are seven areas of grand challenge that are being addressed by use of the methodologies of biological engineering. There exist substantial similarities of tools and approaches utilized across highly disparate applications. All of these challenges require what is often referred to as interdisciplinary research and development, however, all lie under the broad umbrella of biological engineering. The solutions suggested above are typically scientific that offers the critical starting points. The scale-up of these solutions to the benefit of society as a whole require cost and benefit analysis. These solutions also provide unique opportunities in interdisciplinary research. For example, chemical and bioprocess engineers can help design a CO_2 _capture - algae growth system; systems engineers and mathematicians can provide modeling expertise on managing the nitrogen cycle; civil and environmental engineers are essential in providing clean water; computer scientists' collaboration is expected for health informatics; pharmacologists and chemists are needed to engineer better medicines; and biochemists, mathematicians, and systems engineers can help develop synthetic biology tools.

We have not yet described how technology impacts the NAE goal and human concern for joy of living. Certainly the presence and preservation of the natural beauty of our environment, the attainment of health and vitality, and the pleasures of a good meal merit our largest efforts to promote and promulgate the field of biological engineering.

## Competing interests

The authors declare that they have no competing interests.

## Authors' contributions

JYY and MRR jointly conceived the idea and wrote the manuscript.
